# Oxidative stress in susceptibility to breast cancer: study in Spanish population

**DOI:** 10.1186/1471-2407-14-861

**Published:** 2014-11-21

**Authors:** Patricia Rodrigues, Griselda de Marco, Jessica Furriol, Maria Luisa Mansego, Mónica Pineda-Alonso, Anna Gonzalez-Neira, Juan Carlos Martin-Escudero, Javier Benitez, Ana Lluch, Felipe J Chaves, Pilar Eroles

**Affiliations:** INCLIVA Biomedical Research Institute, Valencia, Spain; Genotyping and Genetic Diagnosis Unit, Hospital Clínico Universitario de Valencia INCLIVA Biomedical Research Institute, Valencia, Spain; Internal Medicine, Hospital Rio Hortega, University of Valladolid, Valladolid, Spain; Human Genetics Group, Spanish National Cancer Centre (CNIO) and Biomedical Network on Rare Diseases (CIBERER), Madrid, Spain; Department of Haematology and Medical Oncology, Hospital Clínico Universitario de Valencia, Valencia, Spain; Genotyping Unit, CEGEN, Spanish National Cancer Center (CNIO), Madrid, Spain; Clinical Institute 1 CCBIO, University of Bergen, Bergen, Norway; Department of Nutrition and Food Sciences, Physiology and Toxicology, University of Navarra, Pamplona, Spain

**Keywords:** Breast cancer, Oxidative stress, Single nucleotide polymorphisms, Gene-gene interactions, Multifactor dimensionality reduction

## Abstract

**Background:**

Alterations in the redox balance are involved in the origin, promotion and progression of cancer. Inter-individual differences in the oxidative stress regulation can explain a part of the variability in cancer susceptibility.

The aim of this study was to evaluate if polymorphisms in genes codifying for the different systems involved in oxidative stress levels can have a role in susceptibility to breast cancer.

**Methods:**

We have analyzed 76 single base polymorphisms located in 27 genes involved in oxidative stress regulation by SNPlex technology. First, we have tested all the selected SNPs in 493 breast cancer patients and 683 controls and we have replicated the significant results in a second independent set of samples (430 patients and 803 controls). Gene-gene interactions were performed by the multifactor dimensionality reduction approach.

**Results:**

Six polymorphisms rs1052133 (*OGG1*), rs406113 and rs974334 (*GPX6*), rs2284659 (*SOD3*), rs4135225 (*TXN*) and rs207454 (*XDH*) were significant in the global analysis. The gene-gene interactions demonstrated a significant four-variant interaction among rs406113 (*GPX6*), rs974334 (*GPX6*), rs105213 (*OGG1*) and rs2284659 (*SOD3*) (p-value = 0.0008) with high-risk genotype combination showing increased risk for breast cancer (OR = 1.75 [95% CI; 1.26-2.44]).

**Conclusions:**

The results of this study indicate that different genotypes in genes of the oxidant/antioxidant pathway could affect the susceptibility to breast cancer. Furthermore, our study highlighted the importance of the analysis of the epistatic interactions to define with more accuracy the influence of genetic variants in susceptibility to breast cancer.

## Background

Despite breast cancer (BC) being the most frequent cancer in women in western countries and the second cause of cancer death after lung cancer [1], the risk factors that lead to the disease are not completely understood, although is widely accepted that they include a combination of environmental and genetic factors. For genetic approximation, a polygenic model has been proposed in which a combination of common variants, having individually a modest effect, together contribute to BC predisposition [2].

Numerous evidence links carcinogenesis and oxidative stress regulation, including prooxidant and antioxidant defense systems [3–7]. Oxidative stress is defined as an imbalance in the production of reactive oxygen species (ROS) and reactive nitrogen species (RNS) and their removal by antioxidants. When this imbalance occurs, biomolecules are damaged by ROS and RNS and normal cellular metabolism is impaired, leading to changes of intra- and extracellular environmental conditions. ROS can cause lesions in DNA, such as mutations, deletions, gene amplification and rearrangements, that may lead to malignant transformations and cancer initiation and progression [8–10]. The effect of ROS and RNS, however, is balanced by the anti-oxidant action of non-enzymatic and anti-oxidant enzymes maintaining cellular redox levels under physiological conditions [4, 11].

Previous studies with knockout animals that lack antioxidant enzymes support the view that ROS contribute to the age-related development of cancer. For instance, mice deficient in the antioxidant enzyme CuZnSOD showed increased cell proliferation in the presence of persistent oxidative damage contributing to hepatocarcinogenesis later in life [12]. Another study showed that mice lacking the antioxidant enzyme Prdx1 had a shortened lifespan owing to the development, beginning at about 9 months, of severe hemolytic anemia and several malignant cancers [13].

In this context, single nucleotide polymorphisms (SNPs) in components of the cellular redox systems can modify the redox balance and take part in both the BC initiation and/or progression, as well as determine possible therapeutic treatments [14–17].

Despite the importance of oxidative stress in the development and progression of cancer, few studies have evaluated the relationship between genetic modification in genes coding for enzymes relatives to the redox system and the susceptibility to develop BC. The previous studies had focused mainly on the analysis of genes related to antioxidant defense enzymes [18, 19], but the information about modifications in genes involved in the oxidation process is relatively sparse.

The aim of this study was to evaluate the association between common variants in genes coding for proteins related to the redox system (antioxidant and oxidant systems or proteins) and the susceptibility to develop BC. We hypothesized that common SNPs related to the redox pathway are associated with an altered risk for BC. We chose 76 SNPs on which to perform a two-step study: one first exploratory set and a second, independent, validation set. We also decided to investigate the impact of complex interactions between SNPs at different genes of the stress oxidative pathway. To address this issue, we analyzed the effects of gene-gene interactions by the multifactor dimensionality reduction (MDR) approach. This analysis was carried out in four SNPs that were statistically significant in the combinatorial set.

## Methods

### Study population

The underlying analyses were carried out in a Caucasian Spanish population. The study was carried out in two steps with two population groups. A first group of 1176 samples was composed of 493 female patients diagnosed for BC between the years 1998–2008 at La Paz Hospital and Foundation Jimenez Díaz (Madrid), and 683 healthy women controls recruited at the Hospital of Valladolid (Spain).

Thereupon, we chose the polymorphisms that showed marginally significant association (p-value < = 0.15), and we replicated the procedure in a second independent group (n = 1233) where we included 430 female patients diagnosed for BC between the years 1988–1998 at the Clinic Hospital of Valencia (Spain) and 803 samples from cancer-free women recruited at the blood donor bank at the same Hospital. Blood was collected between 2010 and 2011 during periodical patient visits. The blood from controls was extracted between the years 2009 and 2012. In both groups, the controls were women without pathology or history of cancer. Controls were not matched to cases, but were similar in age. In group 1, cases’ mean age was 57.5 (range 23.5-89.5), and that for donors was 52.7 (21.5-96.5). In group 2, cases’ mean age was 54.1 (20.5-86.5) while in donors, it was 54 (22.5-92.5).

We selected this staged approach because it allowed us to analyze only those polymorphisms with indicative results and reduced the number of genotyping reactions without significantly affecting statistical power [18, 20].

The research protocols were approved by the ethics committee of the INCLIVA Biomedical Research Institute. All the participants in the study were informed and gave their written consent to participate in the study.

### Single nucleotide polymorphisms selection and genotyping

Two public databases were used to collect information about SNPs in oxidative pathway genes: NCBI (http://www.ncbi.nlm.nih.gov/projects/SNP/) and HapMap (http://www.hapmap.org). The selection of polymorphisms was performed by SYSNP [20] and by a literature search in PubMed, Scopus and EBSCO databases using the terms “breast cancer and polymorphisms and oxidative”, along with additional terms such as “SNPs and oxidative pathway and susceptibility”, and their possible combinations. The following criteria were used to select the SNPs: functional known or potentially functional effect, location in promoter regions, minor allele frequency (MAF) over 0.1 in Caucasian populations analyzed previously, localization and distribution along the gene (including upstream and downstream regions) and low described linkage disequilibrium between candidate polymorphisms. We included variants with potential influence in the gene and protein function, as well as the most important variants described in the literature.

Finally, we select a total of 76 polymorphisms located in 27 genes related to the redox system: 17 were classified as antioxidant genes (*CAT*, *GCLC*, *GCLM*, *GNAS*, *GPX6*, *GSR*, *GSS*, *M6PR*, *MSRB2*, *OGG1*, *SOD1*, *SOD2*, *SOD3*, *TXN*, *TXN2*, *TXNRD1*, *TXNRD2*) and 10 as reactive species generators (mainly NADPH oxidase-related genes *CYBB*, *NCF2*, *NCF4*, *NOS1*, *NOS2A*, *NOX1*, *NOX3*, *NOX4*, *NOX5* and *XDH*). Reference names and characteristics of the selected SNPs are provided in Table 1.

**Table 1:** **Summary of the 76 selected SNPs in 27 genes**

Gene	Chr	SNP id	Alleles ^a^	Chr position	Location	MAF controls ^b^	HWE controls ^c^
***CAT***	11	**rs1049982**	**C**/T	34417117	5´UTR	0.355	0.66
		**rs475043**	**A**/G	34450377	downstream	0.334	0.73
		**rs511895**	A/**G**	34444305	intronic	0.332	0.73
		**rs7104301**	**A**/G	34450214	downstream	0.308	0.18
		**rs769214**	**A**/G	34416293	promoter	0.349	0.61
***CYBB***	X	**rs5964125**	**A**/G	37543395	intronic	0.150	0.37
		**rs5964151**	G/**T**	37555673	3´UTR	0.149	0.45
***GCLC***	6	**rs1014852**	**A**/T	53478711	intronic	0.057	0.27
		**rs11415624**	**-**/A	53470407	3´UTR	0.315	0.08
		**rs3736729**	**A**/C	53487364	intronic	0.462	0.36
		**rs4140528**	**C**/T	53469648	downstream	0.265	0.62
***GCLM***	1	**rs7515191**	A/**G**	94139685	intronic	0.368	0.57
		**rs7549683**	**G**/T	94126037	3´UTR	0.367	0.46
***GNAS***	20	**rs4812042**	**A**/G	56895310	intronic	0.340	0.20
		**rs7121**	C/**T**	56912202	coding (synonymous)	0.449	0.70
		**rs919196**	C/**T**	56917480	intronic	0.195	0.63
***GPX6***	6	**rs406113**	**A**/C	28591461	coding (missense)	0.310	0.08
		**rs974334**	**C**/G	28582197	intronic	0.157	0.31
***GSR***	8	**rs1002149**	**G**/T	30705280	promoter	0.152	0.65
		**rs2551715**	**A**/G	30666178	intronic	0.375	1.00
		**rs2911678**	**A**/T	30659513	intronic	0.189	0.53
		**rs8190996**	**C**/T	30673548	intronic	0.437	0.81
***GSS***	20	**rs13041792**	A/**G**	33008716	promoter	0.202	0.63
		**rs2273684**	G/**T**	32993427	intronic	0.438	0.07
		**rs725521**	**C**/T	32979732	downstream	0.456	**0.02**
***M6PR***	12	**rs1805754**	**A**/C	8994515	promoter	0.236	0.16
		**rs933462**	G/**T**	8994932	promoter	0.419	0.94
***MSRB2***	10	**rs11013291**	C/**T**	23440197	intronic	0.394	0.69
***NCF2***	1	**rs2274064**	C/**T**	181809010	coding (missense)	0.444	0.27
		**rs2274065**	**A**/C	181826327	5´UTR	0.067	0.22
		**rs2296164**	**C**/T	181801558	intronic	0.452	0.24
***NCF4***	22	**rs2072712**	**C**/T	35601748	coding (synonymous)	0.089	0.05
***NOS1***	12	**rs570234**	**A**/C	116255365	intronic	0.385	0.80
		**rs576881**	**A**/G	116257218	intronic	0.372	0.62
		**rs816296**	A/**C**	116255127	intronic	0.189	0.17
***NOS2A***	17	**rs2779248**	C/**T**	23151959	upstream	0.362	0.11
		**rs3729508**	**A**/G	23133157	intronic	0.447	0.58
***NOX1***	X	**rs4827881**	**A**/C	100016329	upstream	0.223	0.05
		**rs5921682**	**A**/G	100017093	upstream	0.459	0.88
***NOX3***	6	**rs231954**	C/**T**	155791727	coding (synonymous)	0.442	**0.02**
		**rs3749930**	**G**/T	155802938	coding (missense)	**0.037**	0.24
***NOX4***	11	**rs490934**	C/**G**	88863264	intronic	0.056	1.00
***NOX5***	15	**rs2036343**	**A/**C	67092815	promoter	**0.048**	0.19
		**rs34990910**	A**/G**	67118435	intronic	**0.027**	0.38
***OGG1***	3	**rs1052133**	**C**/G	9773773	coding (missense)	0.213	0.87
***SOD1***	21	**rs17881274**	C/**T**	31953051	upstream	**0.039**	0.07
***SOD2***	6	**rs2842980**	**A**/T	160020106	downstream	0.219	0.50
		**rs2855116**	G/**T**	160026115	intronic	0.454	0.12
		**rs8031**	A/**T**	160020630	intronic	0.459	0.35
***SOD3***	4	**rs2284659**	**G**/T	24403895	promoter	0.371	0.25
***TXN***	9	**rs2301241**	C/**T**	112059329	promoter	0.514	0.25
		**rs4135168**	**A**/G	112056706	intronic	0.222	0.82
		**rs4135179**	**A**/G	112055821	intronic	0.156	0.24
		**rs4135225**	C/**T**	112046512	intronic	0.390	0.29
***TXN2***	22	**rs2281082**	**G**/T	35202696	intronic	0.170	0.41
		**rs5756208**	**A**/T	35207988	promoter	0.179	0.51
***TXNRD1***	12	**rs10861201**	A/**C**	103243089	intronic	0.259	0.31
		**rs4077561**	**C**/T	103204498	promoter	0.387	0.46
		**rs4964287**	**C**/T	103233689	coding (synonymous)	0.320	0.93
		**rs4964778**	**C**/G	103210194	intronic	0.184	0.61
		**rs4964779**	C/**T**	103218991	intronic	0.062	0.31
		**rs5018287**	A/**G**	103231281	intronic	0.419	0.88
***TXNRD2***	22	**rs737866**	**A**/G	18310109	intronic	0.293	0.77
***XDH***	2	**rs10175754**	C/**T**	31475102	intronic	0.153	0.65
		**rs10187719**	**C/**T	31453650	intronic	0.311	0.78
		**rs1346644**	**C**/G	31479549	intronic	0.150	0.55
		**rs1429374**	A/**G**	31425902	intronic	0.338	0.10
		**rs17011353**	**C**/T	31441941	intronic	**0.028**	**0.01**
		**rs17011368**	C/**T**	31444421	coding (missense)	**0.044**	0.54
		**rs17323225**	C/**T**	31446769	coding (missense)	**0.029**	1.00
		**rs1884725**	A/**G**	31425290	coding (synonymous)	0.234	0.20
		**rs206801**	**C**/T	31482250	intronic	0.050	1.00
		**rs206812**	**A**/G	31491373	promoter	0.486	0.54
		**rs2073316**	A/**G**	31464533	intronic	0.427	0.69
		**rs207454**	**A**/C	31421136	intronic	0.087	0.81
		**rs761926**	**C**/G	31444289	intronic	0.300	0.72

Chr – chromosome; MAF – Minor Allele Frequency; HWE – Hardy Weinberg Equilibrium. ^a^majority allele are in bold; ^b^polymorphisms with MAF <5% are excluded for further analysis; ^c^polymorphisms with p-values <0.05 are not in HWE and they are excluded for further analysis. The information about MAF and HWE are referent to the Set 1.

*CAT*: catalase; *CYBB*: cytochrome b-245, beta polypeptide; *GCLC*: glutamate-cysteine ligase, catalytic subunit; *GCLM*: glutamate-cysteine ligase, modifier subunit; *GNAS*: GNAS complex locus; *GPX6*: glutathione peroxidase 6; *GSR*: glutathione reductase; *GSS*: glutathione synthetase; *M6PR*: mannose-6-phosphate receptor; *MSRB2*: methionine sulfoxide reductase B2; *NCF2*: neutrophil cytosolic factor 2; *NCF4*: neutrophil cytosolic factor 4; *NOS1*: nitric oxide synthase 1; *NOS2A*: nitric oxide synthase 2; *NOX1*: NADPH oxidase 1; *NOX3*: NADPH oxidase 3; *NOX4*: NADPH oxidase 4; *NOX5*: NADPH oxidase 5; *OGG1*: 8-oxoguanine DNA glycosylase; *SOD1*: superoxide dismutase 1; *SOD2*: superoxide dismutase 2; *SOD3*: superoxide dismutase 3; *TXN*: thioredoxin; *TXN2*: thioredoxin 2; *TXNRD1*: thioredoxin reductase 1; *TXNRD2*: thioredoxin reductase 2; *XDH*: xanthine dehydrogenase.

### Experimental procedures

The blood samples remained frozen until the DNA extraction was performed. Genomic DNA was extracted from blood samples using DNA Isolation Kit (Qiagen, Izasa, Madrid, Spain) following the manufacturer’s protocol, but a final elution volume of 100 μl used. DNA concentration and quality were measured in a NanoDrop spectrophotometer. Each DNA sample was stored at -20°C until analysis, which in all cases was performed within a year of the DNA extraction.

Genotyping analysis in both sets was performed by SNPlex technology (Applied Biosystems, Foster City, California, USA) according to the manufacturer’s protocol [21]. This genotyping system, based on oligation assay/polymerase chain reaction and capillary electrophoresis, was developed for accurate genotyping, high sample throughput, design flexibility and cost efficiency. It has validated its precision and concordance with genotypes analyzed using TaqMan probes-based assays. The sets of SNPlex probes were reanalyzed in about 10% of the samples with a reproducibility of over 99%. Those polymorphisms and samples with genotyping lower than 85% in the first set were excluded from further analysis.

### Statistical and MDR analyses

Statistical analysis was performed using SNPstats software [22], a free web-based tool, which allows the analysis of association between genetic polymorphisms and diseases. The proper analysis of these studies can be performed with general purpose statistical packages, but this software facilitates the integration of data. The association with disease is modeled as binary; the application assumes an unmatched case–control design and unconditional logistic regression models are used. The statistical analyses are performed in a batch call to the R package (http://www.R-project.org). SNPStats returns a complete set of results for the analysis. SNPstats provides genotype frequencies, proportions, odds ratios (OR) and 95% confidence intervals (CI), and p-values for multiple inheritance models. The lowest Akaike’s Information Criterion and Bayesian Information Criterion values indicate the best inheritance genetic model for each specific polymorphism. All the analyses were adjusted by age. Only SNPs with no significant deviation from Hardy-Weinberg equilibrium (HWE) in controls and a MAF exceeding 5% were retained for the association analysis (Table 1).

To identify gene-gene interactions, MDR was used. It is a non-parametric and a genetic model-free approach that uses a data reduction strategy [23–25]. This method considers a single variable that incorporates information from several loci that can be divided into high risk and low risk combinations. This new variable can be evaluated for its ability to classify and predict outcome risk status using cross validation and permutation testing. Both were used to prevent over-fitting and false-positives from the multiple testing. With n-fold cross-validation, the data are divided into n equal size pieces. An MDR model is fit using (n-1)/n of the data (the training set) and then evaluated for its generalizability on the remaining 1/n of the data (the testing set). The fitness of a MDR model is assessed by estimating accuracy in the training set and the testing set. Moreover, it estimates the degree to which the same best model is discovered across n divisions of the data, referred to as the cross-validation consistency (CVC). The best MDR model is the one with the maximum testing accuracy. Statistical significance is determined using permutation testing. We used 10-fold cross-validation and 1000-fold permutation testing. MDR results were considered statistically significant at the 0.05 level. The advantages of this method are that there are no underlying assumptions about the independence or biological relevance of SNPs or any other factor. This is important for diseases as sporadic BC where the etiology is not completely known. We used the MDR software (version 2.0 beta 8.4) which is freely available (Epistasis.org: http://www.epistasis.org).

## Results

### Single nucleotide polymorphisms and susceptibility to breast cancer

Set 1: To determine the possible association of polymorphisms related to oxidative stress genes and BC we analyzed 76 polymorphisms in 27 genes of the redox system in 493 cases and 683 controls (Table 1). Seven SNPs (rs3749930, rs2036343, rs34990910, rs17881274, rs17011353, rs17011368, rs17323225) with MAF <0.05 in controls, as along with two SNPs (rs725521 and rs231954) not showing Hardy-Weinberg equilibrium, were excluded from the association analysis (Table 1). A total of 67 SNPs were successfully genotyped and analyzed.

Our association analysis in set 1 pointed out four nominally statistically significant results (p < 0.05). Table 2 shows the results found in the selected polymorphisms. Polymorphisms rs974334, rs1805754 rs4135225 and rs207454 showed an association with modifications in the risk for BC. All the results were adjusted by age.

**Table 2:** **Comparison of genotype frequencies between breast cancer patients and controls (Set 1)**

SNP name	Genetic model	OR(95%CI)	Genotype	Controls(n = 683)	Patients(n = 493)	p-value*	AIC
rs1049982	Recessive	0.93 (0.63-1.37)	T/T	79 (12.8%)	54 (13.4%)	0.72	1309.4
			C/C-C/T	537(87.2%)	350 (86.6%)		
rs475043	Recessive	1.27 (0.88-1.85)	G/G	75 (11.5%)	62 (13.3%)	0.21	1454.3
			A/A-A/G	577 (88.5%)	404 (86.7%)		
rs511895	Recessive	1.25 (0.86-1.81)	A/A	74 (11.4%)	60(12.9%)	0.25	1451.9
			G/G-A/G	576(88.6%)	404 (87.1%)		
rs7104301	Recessive	0.78 (0.51-1.19)	G/G	69 (10.6%)	40 (8.6%)	0.24	1454.3
			A/A-A/G	584 (89.4%)	425 (91.4%)		
rs769214	Recessive	0.86 (0.59-1.26)	G/G	80 (12.5%)	57 (12.4%)	0.45	1436
			A/A-A/G	560 (87.5%)	404 (87.6%)		
rs5964125	Dominant	1.07 (0.81-1.40)	A/G-G/G	182 (27.8%)	130 (27.8%)	0.65	1462.2
			A/A	472 (72.2%)	337 (72.2%)		
rs5964151	Dominan	1.10 (0.83-1.44)	G/T-G/G	181 (27.7%)	133 (28.5%)	0.51	1459.3
			T/T	472 (72.3%)	333 (71.5%)		
rs1014852	Recessive	1.22 (0.29-5.19)	T/T	4 (0.6%)	4 (0.9%)	0.79	1462.3
			A/A-A/T	650 (99.4%)	463 (99.1%)		
rs11415624	Recessive	0.90 (0.60-1.34)	A/A	73 (11.2%)	47 (10.1%)	0.59	1458.5
			D/D-D/A	579 (88.8%)	419 (89.9%)		
rs3736729	Recessive	0.74 (0.54-1.01)	C/C	147 (22.4%)	79 (16.9%)	0.058**	1458.7
			A/A-A/C	508 (77.6%)	387 (83.1%)		
rs4140528	Dominant	0.98 (0.77-1.25)	C/T-T/T	297 (45.6%)	206 (44.4%)	0.87	1455.4
			C/C	354 (54.4%)	258 (55.6%)		
rs7515191	Recessive	1.21 (0.86-1.70)	A/A	93 (14.2%)	77 (16.5%)	0.22	1461.7
			G/G-A/G	562 (85.8%)	390 (83.5%)		
rs7549683	Recessive	1.21 (0.86-1.70)	T/T	93 (14.3%)	76 (16.3%)	0.27	1454.8
			G/G-G/T	556 (85.7%)	391 (83.7%)		
rs4812042	Dominant	0.96 (0.75-1.23)	A/G-G/G	357 (55.6%)	258 (55.4%)	0.73	1449.3
			A/A	285 (44.4%)	208 (44.6%)		
rs7121	Recessive	0.97 (0.71-1.32)	C/C	130 (20.3%)	93 (19.9%)	0.84	1442.5
			T/T-C/T	509 (79.7%)	374 (80.1%)		
rs919196	Dominant	1.12 (0.87-1.45)	C/T-C/C	224 (34.3%)	169 (36.2%)	0.37	1460.2
			T/T	429 (65.7%)	298 (63.8%)		
rs406113	Dominant	1.26 (0.98-1.62)	A/C-C/C	314 (51%)	272 (57.6%)	0.066**	1408.9
			A/A	302 (49%)	200 (42.4%)		
rs974334	Recessive	**2.01** **(1.07** **-3.80)**	G/G	18 (2.8%)	25 (5.4%)	0.03**	1452.9
			C/C-C/G	633 (97.2%)	441 (94.6%)		
rs1002149	Dominant	0.95 (0.72-1.26)	G/T-T/T	173 (28.2%)	127 (27.4%)	0.73	1417.4
			G/G	441 (71.8%)	337 (72.6%)		
rs2551715	Recessive	0.88 (0.62-1.26)	A/A	94 (14.4%)	60 (12.9%)	0.5	1455.5
			G/G-A/G	557 (85.6%)	405 (87.1%)		
rs2911678	Recessive	0.96 (0.48-1.95)	T/T	20 (3.1%)	14 (3%)	0.92	1443.5
			A/A-A/T	629 (96.9%)	445 (97%)		
rs8190996	Recessive	1.22 (0.89-1.66)	T/T	126 (19.4%)	96 (20.6%)	0.21	1456.6
			C/C-C/T	524 (80.6%)	371 (79.4%)		
rs13041792	Dominant	1.13 (0.88-1.45)	A/G-A/A	240 (36.9%)	181 (39.1%)	0.35	1450.9
			G/G	411 (63.1%)	282 (60.9%)		
rs2273684	Recessive	1.21 (0.88-1.65)	G/G	115 (17.6%)	92 (19.8%)	0.24	1456
			T/T-G/T	539 (82.4%)	373 (80.2%)		
rs1805754	Dominant	**1.31** **(1.02-** **1.68)**	A/C-C/C	257 (40%)	215 (46.1%)	0.034**	1444.2
			A/A	386 (60%)	251 (53.9%)		
rs933462	Dominant	1.06 (0.82-1.38)	G/T-G/G	431 (66.6%)	317 (68%)	0.64	1452.2
			T/T	216 (33.4%)	149 (32%)		
rs11013291	Recessive	1.09 (0.79-1.51)	C/C	106 (16.3%)	82 (17.6%)	0.6	1458.5
			T/T-C/T	546 (83.7%)	385 (82.4%)		
rs2274064	Recessive	0.88 (0.65-1.20)	C/C	136 (21.7%)	91 (20%)	0.42	1413.4
			T/T-C/T	491 (78.3%)	364 (80%)		
rs2274065	Dominant	1.21 (0.85-1.72)	A/C-C/C	84 (12.9%)	69 (14.8%)	0.3	1458.9
			A/A	568 (87.1%)	398 (85.2%)		
rs2296164	Recessive	0.84 (0.62-1.14)	T/T	143 (22.6%)	91 (19.7%)	0.25	1434.1
			C/C-C/T	491 (77.4%)	370 (80.3%)		
rs2072712	Dominant	1.16 (0.84-1.59)	C/T-T/T	108 (16.5%)	88 (18.9%)	0.37	1461.4
			C/C	547 (83.5%)	378 (81.1%)		
rs570234	Dominant	1.03 (0.79-1.34)	A/C-C/C	388 (63.2%)	273 (63%)	0.81	1355.7
			A/A	226 (36.8%)	160 (37%)		
rs576881	Dominant	1.13 (0.88-1.46)	A/G-G/G	390 (60.6%)	293 (62.9%)	0.33	1448.1
			A/A	254 (39.4%)	173 (37.1%)		
rs816296	Dominant	1.20(0.93-1.55)	A/C-A/A	211 (32.5%)	172 (36.8%)	0.17	1455.6
			C/C	438 (67.5%)	295 (63.2%)		
rs2779248	Recessive	0.88 (0.60-1.29)	C/C	75 (11.7%)	55 (11.8%)	0.52	1444.5
			T/T-C/T	569 (88.3%)	410 (88.2%)		
rs3729508	Dominant	0.90 (0.69-1.18)	A/G-A/A	443 (70.4%)	316 (69%)	0.46	1421.5
			G/G	186 (29.6%)	142 (31%)		
rs4827881	Dominant	1.00 (0.78-1.29)	A/C-A/A	246 (37.6%)	177 (37.9%)	0.98	1462.8
			C/C	408 (62.4%)	290 (62.1%)		
rs5921682	Dominant	1.12 (0.85-1.47)	A/G-G/G	459 (70.4%)	339 (72.6%)	0.42	1459.8
			A/A	193 (29.6%)	128 (27.4%)		
rs490934	Recessive	3.34 (0.57-19.43)	C/C	2 (0.3%)	4 (0.9%)	0.16	1459.6
			G/G-C/G	651 (99.7%)	463(99.1%)		
rs1052133	Recessive	1.76 (1.00-3.10)	G/G	34 (5.1%)	28 (8.1%)	0.051**	1124
			C/C-C/G	631 (94.9%)	319 (91.9%)		
rs2842980	Recessive	1.40 (0.82-2.37)	T/T	31 (4.9%)	31 (6.7%)	0.22	1439.2
			A/A-A/T	606 (95.1%)	435 (93.3%)		
rs2855116	Dominant	0.89 (0.68-1.16)	G/T-G/G	440 (69.3%)	313 (67%)	0.39	1444.1
			T/T	195 (30.7%)	154 (33%)		
rs8031	Dominant	0.85 (0.65-1.10)	A/T-A/A	458 (70.6%)	313 (67.2%)	0.22	1454.3
			T/T	191 (29.4%)	153 (32.8%)		
rs2284659	Recessive	1.30 (0.92-1.84)	T/T	83 (12.9%)	80 (17.2%)	0.14**	1445.6
			G/G-G/T	560 (87.1%)	386 (82.8%)		
rs2301241	Dominant	0.80 (0.60-1.07)	C/T-C/C	460 (74.4%)	348 (71.2%)	0.14**	1350.5
			T/T	158 (25.6%)	141 (28.8%)		
rs4135168	Dominant	1.17 (0.90-1.52)	A/G-G/G	238 (39.9%)	183 (43.4%)	0.23	1328
			A/A	359 (60.1%)	239 (56.6%)		
rs4135179	Dominant	1.27 (0.97-1.66)	A/G-G/G	178 (28%)	151 (32.8%)	0.085**	1431
			A/A	458 (72%)	310 (67.2%)		
rs4135225	Recessive	**0.66** **(0.45-** **0.96)**	C/C	104 (16.2%)	48 (10.5%)	0.029**	1431.1
			T/T-C/T	536 (83.8%)	410 (89.5%)		
rs2281082	Recessive	1.52 (0.73-3.17)	T/T	15 (2.3%)	16 (3.5%)	0.26	1443.5
			G/G-G/T	627 (97.7%)	447 (96.5%)		
rs5756208	Recessive	1.54 (0.77-3.11)	T/T	17 (2.7%)	17 (3.7%)	0.23	1438.5
			A/A-A/T	618 (97.3%)	445 (96.3%)		
rs10861201	Recessive	0.69 (0.41-1.17)	A/A	49 (7.6%)	23 (5.1%)	0.16	1411.8
			C/C-A/C	592 (92.4%)	425 (94.9%)		
rs4077561	Recessive	1.20 (0.86-1.68)	T/T	92 (14.6%)	80 (17.4%)	0.28	1425
			C/T-T/T	540 (85.4%)	380 (82.6%)		
rs4964287	Dominant	1.09 (0.85-1.40)	C/T-T/T	356 (54.4%)	266 (57%)	0.48	1461.9
			C/C	298 (45.6%)	201 (43%)		
rs4964778	Dominant	1.17 (0.90-1.51)	C/G-G/G	216 (33.1%)	171 (36.7%)	0.24	1457.3
			C/C	436 (66.9%)	295 (63.3%)		
rs4964779	Dominant	1.16 (0.80-1.68)	C/T-C/C	76 (11.8%)	62 (13.3%)	0.43	1451.5
			T/T	569 (88.2%)	405 (86.7%)		
rs5018287	Recessive	1.12 (0.82-1.53)	A/A	118 (18%)	91 (19.6%)	0.47	1453.9
			G/G-A/G	536 (82%)	372 (80.4%)		
rs737866	Dominant	1.16 (0.90-1.49)	A/G-G/G	298 (49%)	234 (53.4%)	0.25	1364.1
			A/A	310 (51%)	204 (46.6%)		
rs10175754	Dominant	0.92 (0.69-1.22)	C/T-C/C	177 (27.8%)	118 (26%)	0.55	1418.6
			T/T	460 (72.2%)	335 (74%)		
rs10187719	Recessive	0.80 (0.51-1.26)	T/T	59 (9.9%)	34 (7.9%)	0.34	1353.8
			C/C-C/T	539 (90.1%)	398 (92.1%)		
rs1346644	Recessive	1.48 (0.71-3.09)	G/G	16 (2.5%)	15 (3.2%)	0.29	1458
			C/C-C/G	637 (97.5%)	450 (96.8%)		
rs1429374	Dominant	1.15 (0.89-1.47)	A/G-A/A	356 (54.9%)	274 (59%)	0.28	1450.4
			G/G	292 (45.1%)	190 (41%)		
rs1884725	Recessive	0.71 (0.42-1.22)	A/A	42 (6.5%)	23 (5%)	0.21	1444.1
			G/G-A/G	604 (93.5%)	439 (95%)		
rs206801	Recessive	3.42 (0.33-35.82)	T/T	1 (0.2%)	3 (0.6%)	0.27	1462
			C/C-C/T	654 (99.8%)	464 (99.4%)		
rs206812	Recessive	0.96 (0.72-1.28)	A/A	159 (24.4%)	111 (23.8%)	0.78	1461.8
			G/G-A/G	494 (75.6%)	356 (76.2%)		
rs2073316	Recessive	1.14 (0.84-1.56)	A/A	118 (18.6%)	95 (20.8%)	0.4	1423.7
			G/G-A/G	517 (81.4%)	361 (79.2%)		
rs207454	Recessive	**4.98** **(1.28-** **19.34)**	C/C	3 (0.5%)	9 (1.9%)	0.012**	1450.3
			A/A-A/C	646 (99.5%)	457 (98.1%)		
rs761926	Dominant	0.85 (0.66-1.08)	C/G-G/G	334 (51.1%)	214 (45.8%)	0.18	1459.4
			C/C	319 (48.9%)	253 (54.2%)		

CI, confidence interval; OR, odds ratio. *p-values adjusted by age. In bold p-values <0.05. **polymorphisms with a p-value<=0.15. Set 1 (n=1176; cases=493 and controls=683). The best model have been chosen with the criteria of lower AIC (Akaike information criterion) and lower BIC (Bayesian information criterion) values. Only AIC is shown in table.

Set 2: Subsequently, and in order to better identify those polymorphisms that could be associated with BC, we replicated the 10 SNPs with a *p*-*value* equal to or lower than 0.15 in group 1 [rs3736729, OR: 0.74 (0.54-1.01); rs406113, OR:1.26 (0.98-1.62); rs974334, OR:2.01 (1.07-3.80); rs1805754, OR: 1.31 (1.02-1.68); rs1052133, OR: 1.76 (1.00-3.10); rs2284659, OR: 1.30 (0.92-1.84); rs2301241, OR: 0.80 (0.60-1.07); rs4135179, OR: 1.27 (0.97-1.66); rs4135225, OR: 0.66 (0.45-0.96); rs207454, OR: 4.98 (1.28-19.34)] in a second independent set.

Set 1 + Set 2: Finally, we analyzed the 10 polymorphisms in the global population set 1 + set 2 (n = 2409; cases = 923, controls = 1486). The results are listed in Table 3. From the 10 polymorphisms analyzed in both samples, 6 presented a statistically significant association with increased risk when the combined data were analyzed: rs406113 [OR: 1.23 (1.04-1.46)], rs974334 [OR: 1.73 (1.09-2.73)], rs1052133 [OR:1.82 (1.31-2.52)], rs2284659 [OR:1.33 (1.05-1.67), rs4135225 [OR: 0.77 (0.60-0.99)], rs207454 [OR: 2.12 (1.11-4.04)]. Of these polymorphisms, the rs105213 on the *OGG1* gene maintained the statistical significance (p-value = 0.0004) after the Bonferroni correction.

**Table 3:** **Genotype frequencies of relevant polymorphisms in different Sets**

Gene SNP name	Set	Genetic Model	OR(95%CI)	Genotype	Controls	Patients	p-value	AIC
**GCLC** rs3736729	Set 1	Recessive	0.74 (0.54-1.01)	C/C	147 (22.4%)	79 (16.9%)	0.058	1458.7
				A/A-A/C	508 (77.6%)	387 (83.1%)		
	Set 2	Recessive	0.89 (0.67-1.19)	C/C	191 (23.9%)	89 (21.8%)	0.43	1549.3
				A/A-A/C	610 (76.2%)	319 (78.2%)		
	Set 1 + 2	Recessive	0.85 (0.73-1.00)	C/C	343 (23.1%)	177 (19.7%)	0.054	3160.5
				A/A-A/C	1141 (76.9%)	723 (80.3%)		
**GPX6** rs406113	Set 1	Dominant	1.26 (0.98-1.62)	A/C-C/C	314 (51%)	272 (57.6%)	0.066	1408.9
				A/A	302 (49%)	200 (42.4%)		
	Set 2	Dominant	1.18 (0.93-1.50)	A/C-C/C	435 (54.2%)	241 (58.4%)	0.17	1559.7
				A/A	367 (45.8%)	172 (41.6%)		
	Set 1 + 2	Dominant	**1.23** **(1.04-** **1.46)**	A/C-C/C	759 (52.7%)	524 (57.8%)	**0.015**	3127.7
				A/A	681 (47.3%)	382 (42.2%)		
**GPX6** rs974334	Set 1	Recessive	**2.01** **(1.07-** **3.80)**	G/G	18 (2.8%)	25 (5.4%)	**0.03**	1452.9
				C/C-C/G	633 (97.2%)	441 (94.6%)		
	Set 2	Recessive	1.45 (0.70-3.01)	G/G	17 (2.1%)	13 (3%)	0.33	1592.6
				C/C-C/G	785 (97.9%)	415 (97%)		
	Set 1 + 2	Recessive	**1.73** **(1.09** **-2.73)**	G/G	37 (2.5%)	39 (4.2%)	**0.02**	3194.7
				C/C-C/G	1444 (97.5%)	881 (95.8%)		
**M6PR** rs1805754	Set 1	Dominant	**1.31** **(1.02** **-1.68)**	A/C-C/C	257 (40%)	215 (46.1%)	**0.034**	1444.2
				A/A	386 (60%)	251 (53.9%)		
	Set 2	Dominant	1.05 (0.83-1.34)	A/C-C/C	347 (44.1%)	191 (45.4%)	0.67	1565.8
				A/A	440 (55.9%)	230 (54.6%)		
	Set 1 + 2	Dominant	1.15 (0.98-1.36)	A/C-C/C	619 (42.5%)	420 (46%)	0.093	3160.7
				A/A	838 (57.5%)	493 (54%)		
**OGG1** rs1052133	Set 1	Recessive	1.76 (1.00-3.10)	G/G	34 (5.1%)	28 (8.1%)	0.051	1124
				C/C-C/G	631 (94.9%)	319 (91.9%)		
	Set 2	Recessive	**1.84** **(1.11-** **3.07)**	G/G	33 (4.1%)	30 (7.3%)	**0.02**	1543.7
				C/C-C/G	767 (95.9%)	378 (92.7%)		
	Set 1 + 2	Recessive	**1.82** **(1.31-** **2.52)**	G/G	64 (4.5%)	56 (7.9%)	**4e** **-04**	2665.8
				C/C-C/G	1348 (95.5%)	655 (92.1%)		
**SOD3** rs2284659	Set 1	Recessive	1.30 (0.92-1.84)	T/T	83 (12.9%)	80 (17.2%)	0.14	1445.6
				G/G-G/T	560 (87.1%)	386 (82.8%)		
	Set 2	Recessive	1.25 (0.90-1.73)	T/T	109 (13.6%)	69 (16.4%)	0.19	1577
				G/G-G/T	693 (86.4%)	352 (83.6%)		
	Set 1 + 2	Recessive	**1.33** **(1.05-** **1.67)**	T/T	194 (13.2%)	153 (16.8%)	**0.017**	3172.3
				G/G-G/T	1278 (86.8%)	760 (83.2%)		
**TXN** rs2301241	Set 1	Dominant	0.80 (0.60-1.07)	C/T-C/C	460 (74.4%)	348 (71.2%)	0.14	1350.5
				T/T	158 (25.6%)	141 (28.8%)		
	Set 2	Dominant	1.35 (1.03-1.78	C/T-C/C	569 (71.2%)	318 (77%)	**0.03**	1554.4
				T/T	230 (28.8%)	95 (23%)		
	Set 1 + 2	Dominant	1.05 (0.87-1.27)	C/T-C/C	1014 (72.9%)	660 (73.9%)	0.59	3060.6
				T/T	377 (27.1%)	233 (26.1%)		
**TXN** rs4135179	Set 1	Dominant	1.27 (0.97-1.66)	A/G-G/G	178 (28%)	151 (32.8%)	0.085	1431
				A/A	458 (72%)	310 (67.2%)		
	Set 2	Dominant	1.08 (0.83-1.39)	A/G-G/G	248 (31%)	136 (32.5%)	0.57	1571.2
				A/A	553 (69%)	282 (67.5%)		
	Set 1 + 2	Dominant	1.14 (0.95-1.36)	A/G-G/G	435 (29.7%)	294 (32.5%)	0.16	3153
				A/A	1029 (70.3%)	611 (67.5%)		
**TXN** rs4135225	Set 1	Recessive	0.66 (0.45-0.96)	C/C	104 (16.2%)	48 (10.5%)	0.029	1431.1
				T/T-C/T	536 (83.8%)	410 (89.5%)		
	Set 2	Recessive	0.97 (0.68-1.38)	C/C	104 (13%)	53 (12.7%)	0.87	1574.5
				T/T-C/T	698 (87%)	366 (87.3%)		
	Set 1 + 2	Recessive	**0.77** **(0.60-** **0.99)**	C/C	212 (14.4%)	104 (11.5%)	**0.041**	3151.7
				T/T-C/T	1257 (85.6%)	799 (88.5%)		
**XDH** rs207454	Set 1	Recessive	**4.98** **(1.28-** **19.34)**	C/C	3 (0.5%)	9 (1.9%)	**0.012**	1450.3
				A/A-A/C	646 (99.5%)	457 (98.1%)		
	Set 2	Recessive	1.61 (0.54-4.83)	C/C	7 (0.9%)	6 (1.4%)	0.4	1589.1
				A/A-A/C	793 (99.1%)	421 (98.6%)		
	Set 1 + 2	Recessive	**2.12** (**1.11**-**4.04**)	C/C	11 (0.8%)	15 (1.6%)	**0.024**	3188.5
				A/A-A/C	1464 (99.2%)	904 (98.4%)		

Table polymorphisms were chosen from the analysis of the first set of patients with the criteria of a cutoff p-value equal or lower a 0.15. Bold number indicate result statistically significant, p-value<0.05. Set 1 (n = 1176; cases = 493 and controls=683), Set 2 (n = 1233; cases=430 and controls = 803), Set 1 + set 2 (n = 2409; cases = 923 and controls=1486). GCLC: glutamate-cysteine ligase, catalytic subunit; GPX6: glutathione peroxidase 6; M6PR: mannose-6-phosphate receptor; OGG1: 8-oxoguanine DNA glycosylase; SOD3: superoxide dismutase 3; TXN: thioredoxin; XDH: xanthine dehydrogenase.

### Gene-gene interactions in breast cancer patients

There is growing evidence that epistasis interactions between genes may play a role in cancer risk, and different variable selection approaches have been developed to analyze the potential gene-gene and gene-environment interactions [25]. The four most significantly associated polymorphisms in set 1 + set 2 with susceptibility to BC were selected for this analysis: rs406113 [OR: 1.23 (1.04-1.46)], rs974334 [OR: 1.73 (1.09-2.73)], rs1052133 [OR:1.82 (1.31-2.52)] and rs2284659 [OR:1.33 (1.05-1.67)]. Data from 1182 samples (controls and patients) from both groups were used. The combination was performed grouping the genotypes according to the model predicted for the four polymorphisms: recessive model for rs1052133 (CC and CG were grouped into a single block), dominant model for rs406113 (CC and AC genotypes were grouped into a single block), recessive model for rs974334 (CC and CG genotypes were grouped into a single block) and recessive model for rs2284659 (GG and GT genotypes were grouped into a single block). For a two-loci interaction, the combination of polymorphisms rs406113 (*GPX6*) and rs1052133 (*OGG1*) was the most significant (p = 0.041). The best three-loci model included rs406113 on the *GPX6* gene, rs1052133 on the *OGG1* gene and rs2284659 on the *SOD3* gene, and it showed statistical significance (p < 0.0007) with an OR = 1.82 and 95% CI = 1.28-2.58. A four-way interaction found that between rs406113 on the *GPX6* gene, rs974334 on the GPX6 gene, rs1052133 on the *OGG1* gene and rs2284659 on the *SOD3* gene predicts breast cancer with a testing balance accuracy of 0.5267. This four-loci model had a chi-square value of 11.284 (p = 0.0008) and an OR of 1.75 [95% CI = 1.26-2.44]. The four polymorphism combinatory model showed a higher predisposition to BC than the polymorphisms rs406113, rs974334 and rs2284659 did individually (OR_X2_ = 1.23, OR_X3_ = 1.73, OR_X6_ = 1.33) and had values similar to the ones of polymorphism rs1052133 (OR_X5_ = 1.82). The summary of the multi-factor dimensionality results are listed in Table 4.

**Table 4:** **Summary of Multi**-**factor Dimensionality** (**MDR**) **results**

Model	Training accuracy	Testing accuracy	OR(95%CI)	p-value	CVC*
X2	0.5215	0.5093	1.18 (0.93-1.51)	0.1797	8/10
X2 – X5	0.5319	0.5199	1.30 (1.01-1.66)	0.041	8/10
X2 – X5 – X6	0.5353	**0.5210**	1.82 (1.28-2.58)	**0.0007**	7/10
X2 – X3 – X5 – X6	0.5371	**0.5267**	1.75 (1.26-2.44)	**0.0008**	**10/** **10**

The polymorphisms rs406113, rs974334, rs1052133 and rs2284659 showing the highest statistical significance in the combinatorial set1 + set 2 were chosen for gene-gene interaction analysis. *CVC, cross-validation consistency. Testing accuracy, p-value and CVC significant were highlighted in bold.

X2 = rs406113 (*GPX6*), X3 = rs974334 (*GPX6*), X5 = rs1052133 (*OGG1*), X6 = rs2284659 (*SOD3*).

**Figure 1:**
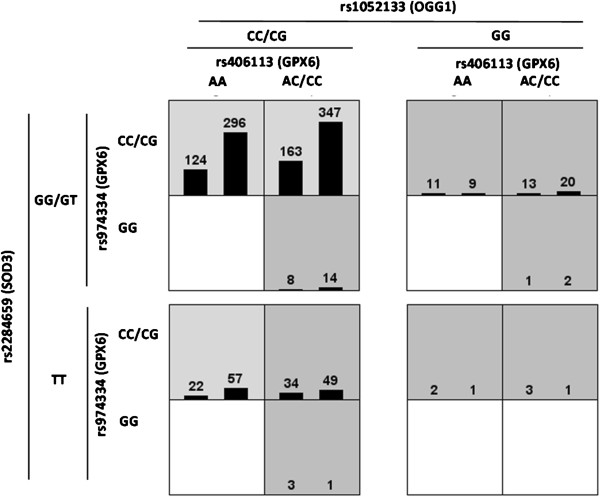
**The polymorphisms rs406113**, **rs974334**, **rs1052133 and rs2284659**, **showing the highest statistical significance in the combinatorial set 1** **+** **set 2**, **were chosen for the gene**-**gene interaction analysis.** The MDR analysis was done with the genotypes collapsed according to the genetic models selected: rs1052133 (*OGG1*) recessive model; CC/CG *vs*.GG, rs406113 (*GPX6*) dominant model; CC/AC *vs*. AA, rs974334 (*GPX6*) recessive model; CC/CG *vs*. GG, rs2284659 (*SOD3*) recessive model; GG/GT *vs*. TT. The figure shows the summary of four-loci genotype combinations associated with high and low risk. Cases: left bars, controls: right bars. The epistatic gene-gene interaction corresponds to the high risk combinations (darkest color).

The combined genotype AA for rs406113, CC/CG for rs974334, CC/CG for rs1052133 and GG/ GT for rs2284659 showed a higher risk for BC, which is consistent with the models described for the polymorphisms individually. Figure 1 summarizes the four-loci genotype combinations associated with high and low risk and with the distribution of cases and controls.

## Discussion

Genetic association studies involving SNPs and their possible interactions have become increasingly important for the study of human diseases. The present study has focused on genes encoding for proteins of the redox system. It is long proven that they are clearly involved in extensive damage to DNA, which in turn leads to gene mutations and, finally, carcinogenesis. The functionality of polymorphisms in relation to oxidative stress has been proven in several cases. For instance, the polymorphism in exon 2 of the superoxide dismutase 2 (*SOD2*) gene A16V (C/T) (rs4880) led to structural alterations in the domain responsible to target the mitochondria, giving a reduction in the antioxidant potential [26]. Furthermore, a functional polymorphism in exon 9 of the *CAT* gene and other polymorphisms in endothelia NO synthase (*eNOs*) that seem relevant for their activity have been documented [26–28]. Therefore, it is clear that a single oligonucleotide modification can lead to structural changes, modifications in the affinity to bind proteins or in activity that may be relevant to the redox system. Our hypothesis was that variations in genes from the stress oxidative pathway, that have shown to have a possible linkage to BC, can be associated with predisposition to this disease. Indeed, genetic variations in these pathways have shown to modify the risk for BC [29].

In the present epidemiological study, we have assessed the effect of 76 SNPs in 27 genes in a case–control study in a Spanish population. Genotype distributions in the controls did not differ significantly from those expected under HWE. The study was performed in two independent sets of patients and controls, first, to select the relevant polymorphisms and second, to check the reproducibility and significance of these preliminary results.

Six SNPs (rs406113 and rs974334 on the glutathione peroxidase 6 (*GPX6*) gene, rs1052133 on the 8-oxoguanine DNA glycosylase (*OGG1*) gene, rs2284659 on the superoxide dismutase 3 (*SOD3*) gene, rs4135225 on the thioredoxin (*TXN*) gene and rs207454 on the xanthine dehydrogenase (*XDH*) gene) are associated with variations in the predisposition to BC.

The rs406113 (c.39 T > G; p.F13L) and rs974334 (c.242-12G > C) polymorphisms on the *GPX6* gene had not been studied previously; in fact, there was no information available in the literature about polymorphisms on the *GPX6* gene even though they can have a functional effect. Genetic variants in other genes of the GPX family have been associated with BC [30–32].

Thioredoxin (*TXN*) is overexpressed in BC, and it is related to tumor grade [33], being a crucial element in redox homeostasis [34]. Studies of polymorphisms in the *TXN* gene, encoding thioredoxin, are few in cancer. Seibold et al. [19], evaluated the influence of common variants on *TXN*, thioredoxin reductase 1 (*TXNRD1*) and thioredoxin 2 (*TXN2*) genes and the risk of BC after menopause, including seven of the SNPs analyzed in our study. Rs2301241 and rs2281082 were not significantly related to BC risk in our study, however, Seibold et al. found a limited association of rs2301241 with BC risk when comparing rare homozygote *vs*. common homozygote. Other studies found a borderline significance [18]. In the case of rs2281082, the borderline association of the Seibold study was not confirm in other publications [19]. In our study population, we found that carriers of one T allele on rs4135225 (c.196-192C > T) were associated with lower risk for BC development (OR = 0.77 [95% CI; 0.60-0.99] p = 0.041). Seidbol et al. found a predisposition to BC for this polymorphisms (OR = 1.22 [95% CI; 1.06-1.41]. One must take into account that the Seidbold study is focused in postmenopausal women, unlike ours. Still, in their analysis, they compared only two of the three possible genotypes (heterozygotes *vs*. common homozygotes). In any case, their results showed borderline significance.

The xanthine dehydrogenase (*XDH*) is an important enzyme involved in the first-pass metabolism of 6-mercaptopurine [35]. Polymorphisms in the *XDH* gene have been related to cancer. The rs1884725 polymorphism has been identify as a genetic variant associated with disease risk and outcomes in multiple myeloma [36]. In our study, one of the thirteen polymorphisms evaluated on this gene showed an association with BC risk. Carriers of one A allele of rs207454 displayed 2.12 times ([95% CI; 1.11-4.04], p = 0.024) more risk to develop the illness than did non-carriers. To our knowledge, there are no studies of these polymorphisms in the literature. The results presented here suggest an association with the development of BC, although further confirmatory studies would be needed to confirm it.

The polymorphism on the *SOD3* gene (rs2284659) analyzed in our study showed a trend to the predisposition for BC in the global analysis. There was no information about this polymorphism in the literature. Other polymorphisms in this gene, like rs2536512 and rs699473, have been associated in BC patients with the incidence of tumor and poorer progression-free survival (PFS) [37]. Moreover, some results suggest that rs699473 may influence brain tumor risk [38].

The variant rs1052133 (Ser326Cys) in the *OGG1* gene has the same tendency to predisposition for BC in both sets, separately and in the combined data set. Concerning this polymorphism, previous studies had conflicting results [39–45]. Three meta-analyses have attempted to summarize the results [39, 41, 46]. In one study, the authors analyzed this polymorphism in relation to several cancers founding only significant association with the risk for lung cancer [46]. The others two meta-analyses are focused on BC, and the results are contradictory. Yuan et al. found an association just in the European population subgroup [41], while Gu et al. did not show any association, even when stratifying the analysis by ethnicity or menopausal status [39]. These differences may have arisen from the different number of studies included in the European group.

We found an increment for the risk to develop BC in the carriers of at least one Ser allele (recessive model) if we consider the sets both separately and together ((OR = 1.82 [95% CI 1.31-2.52]) and p-value = 0.0004). Our results are in concordance with the meta-analysis by Yuan and collaborators that suggests that the hOGG1 326 Cys allele provides a significant protective effect for BC in European women [41]. The importance of this SNP rests in the role of the 8-oxoguanine DNA glycosylase, encoded by *OGG1* [47]. This enzyme can excise the 8-hydroxy-2´-deoxyguanosine (8-OHdG) modifications occurring in the DNA as a result of hydroxyl radical interaction [41, 48, 49]. An incorrect expression of the protein could interfere with the suitable repair of the genetic material. Other polymorphisms in the *OGG1* gene, like rs2304277, and recently described by Osorio et al., have been associated with ovarian cancer risk in *BRCA1* mutation carriers [50]. This data certainly support the importance of genetic changes in the *OGG1* gene in relation to the predisposition to cancer.

The epistatic analysis of the four most significant polymorphisms in relation to the susceptibility to BC was performed with the MDR method. This is a reliable approach that has been widely used [23, 25, 51–54]. The combination was performed grouping the genotypes according to the model predicted for the four polymorphisms in Tables 2 and 3. The result obtained was an OR = 1.75 [95% CI = 1.26-2.44; p-value = 0.0008], a value similar to that obtained for rs1052133 (OR = 1.82 [95% CI = 1.31-2.52; p-value = 0.0004]). The previous study of Cebrian et al. in antioxidant defence enzymes and BC susceptibility has twelve common SNPs with our study. Two showed discrepancies with our data: Rs511895 in the *CAT* gene was not significant in our analysis, but it presented a borderline tendency in the Cebrian et al. study. Moreover, they found a significant difference in genotype distribution between cases and controls in rs4135179 (*TXN*). We, however, were unable to confirm this in our global analysis, although we detected a marginal significance in set 1. The reason for this discrepancy can be found in the population’s characteristics, in the superior age of the population included in the Cebrian study [18].

Our study has several limitations to take into consideration. Firstly, there is no data available about the lifestyle of controls and patients that could be related to oxidative stress, such as diet, exercise and the consumption of tobacco and alcohol. Secondly, polymorphisms that were not explored in our study may affect the risk to develop BC and should be taken into account in the analysis of our data and in further studies. Nevertheless, the association between SNPs and risk for BC is reliable since that power exceeded 95% in all the cases. All samples are from the same country and ethnicity, and the adjustment for age reduces variability.

Additionally, MDR has 80% statistical power to detect true interactions in two-, three-, and four-way gene-gene interactions, even with a small number of cases and controls [24]. Furthermore, several associations detected in these data involved SNPs occurring in non-coding regions. However, variations in the intronic structure have been proposed to influence cancer susceptibility via regulation of gene expression, gene splicing or mRNA stability. It is also possible that these polymorphisms are in linkage disequilibrium with other functional polymorphisms that may affect BC susceptibility.

Despite these considerations, our work, as far as we know, is the largest study in the Spanish population that analyzes the influence of polymorphisms in oxidative genes in susceptibility to BC. Overall, our data, together with that published in the bibliography [18, 19, 29, 37, 41, 45, 55–62], suggest a role of stress-response gene variants in the susceptibility to BC.

## Conclusions

Our results suggest that different genotypes in genes of the oxidant/antioxidant pathway could affect the susceptibility to breast cancer. We have found six polymorphisms in *OGG1*, *GPX6*, *SOD3*, *TXN* and *XDH* genes significantly associated with predisposition to breast cancer. These associations have not been described previously, except for rs1052133 (*OGG1*). Concerning this polymorphism the published results in breast cancer were contradictory, and some authors found only a significant association with the risk of developing lung cancer. We have found an increment in the risk of developing breast cancer in the carriers of at least one Ser allele (recessive model) in concordance with a meta-analysis of breast cancer susceptibility in European women. In this particular case, an incorrect expression of the protein encoded by the *OGG1* gene could interfere with the suitable repair of the genetic material. Furthermore, our study highlighted the importance of the analysis of the epistatic interactions in order to define the influence of genetic variants in susceptibility to breast cancer more precisely. Further studies on the relevance of these and other polymorphisms in the development of breast cancer should be performed.

## Abbreviations

BC: Breast cancer
BRCA1: Breast cancer 1
CI: Confidence interval
CAT: Catalase
CYBB: Cytochrome b-245, beta polypeptide
GCLC: Glutamate-cysteine ligase, catalytic subunit
GCLM: Glutamate-cysteine ligase, modifier subunit
GNAS: GNAS complex locus
GPX6: Glutathione peroxidase 6
GSR: Glutathione reductase
GSS: Glutathione synthetase
HWE: Hardy-Weinberg Equilibrium
MAF: Minor Allele Frequency
MDR: Multifactor dimensionality reduction
M6PR: mannose-6-phosphate receptor
MSRB2: Methionine sulfoxide reductase B2
NCF2: Neutrophil cytosolic factor 2
NCF4: Neutrophil cytosolic factor 4
NOS1: Nitric oxide synthase 1
NOS2A: Nitric oxide synthase 2
NOX1: NADPH oxidase 1
NOX3: NADPH oxidase 3
NOX4: NADPH oxidase 4
NOX5: NADPH oxidase 5
OGG1: 8-oxoguanine DNA glycosylase
OR: Odds ratio
RNS: Reactive Nitrogen Species
ROS: Reactive Oxygen Species
SOD1: Superoxide dismutase 1
SOD2: Superoxide dismutase 2
SOD3: Superoxide dismutase 3
SNPs: Single Nucleotide Polymorphisms
TXN: Thioredoxin
TXN2: Thioredoxin 2
TXNRD1: Thioredoxin reductase 1
TXNRD2: Thioredoxin reductase 2
XDH: Xanthine dehydrogenase.
